# A Risk Score to Predict Short-term Outcomes Following Emergency Department Discharge

**DOI:** 10.5811/westjem.2018.7.37945

**Published:** 2018-08-13

**Authors:** Gelareh Z. Gabayan, Michael K. Gould, Robert E. Weiss, Vicki Y. Chiu, Catherine A. Sarkisian

**Affiliations:** * University of California, Los Angeles, Department of Emergency Medicine, Los Angeles, California; † Kaiser Permanente Southern California, Department of Research and Evaluation, Pasadena, California; ‡ University of California, Los Angeles, Fielding School of Public Health, Department of Biostatistics, Los Angeles, California; § University of California, Los Angeles, Department of Medicine, Los Angeles, California; ¶ Greater Los Angeles Veterans Affairs Healthcare System, Department of Medicine, Los Angeles, California

## Abstract

**Introduction:**

The emergency department (ED) is an inherently high-risk setting. Risk scores can help practitioners understand the risk of ED patients for developing poor outcomes after discharge. Our objective was to develop two risk scores that predict either general inpatient admission or death/intensive care unit (ICU) admission within seven days of ED discharge.

**Methods:**

We conducted a retrospective cohort study of patients age > 65 years using clinical data from a regional, integrated health system for years 2009–2010 to create risk scores to predict two outcomes, a general inpatient admission or death/ICU admission. We used logistic regression to predict the two outcomes based on age, body mass index, vital signs, Charlson comorbidity index (CCI), ED length of stay (LOS), and prior inpatient admission.

**Results:**

Of 104,025 ED visit discharges, 4,638 (4.5%) experienced a general inpatient admission and 531 (0.5%) death or ICU admission within seven days of discharge. Risk factors with the greatest point value for either outcome were high CCI score and a prolonged ED LOS. The C-statistic was 0.68 and 0.76 for the two models.

**Conclusion:**

Risk scores were successfully created for both outcomes from an integrated health system, inpatient admission or death/ICU admission. Patients who accrued the highest number of points and greatest risk present to the ED with a high number of comorbidities and require prolonged ED evaluations.

## INTRODUCTION

The emergency department (ED) is inherently a high-risk setting, and understanding outcomes following the ED visit is difficult. This is mainly due to the inability to track most patient visits after discharge. Knowledge of a risk score for negative outcomes following ED discharge and probabilities of those outcomes for adults discharged from the ED could help ED practitioners better manage patients as well as their discharge plan.

Risk scores have traditionally helped ED practitioners better understand the risks ED patients face when presenting with certain conditions and signs/symptoms.^1^ The objective of this study was to conduct a retrospective cohort analysis and develop a risk score for adults experiencing an inpatient admission or death/intensive care unit (ICU) placement within seven days of ED discharge.

## METHODS

### Study Design

A multisite retrospective cohort study of ED visits was conducted following the Strengthening the Reporting of Observational studies in Epidemiology guidelines.^2^ This study was approved by the institutional review boards of Kaiser Permanente Southern California and the University of California, Los Angeles.

### Setting

We analyzed clinical data from Kaiser Permanente Southern California (KPSC), an integrated health system that provides comprehensive care to over 3.5 million members at 14 medical centers and 197 offices throughout Southern California. There were 13 health system EDs in operation during the study period. All members have very similar healthcare benefits, including coverage of emergency services both within and outside the health system. Members of the health plan are generally representative of the population of Southern California, which is a racially and socioeconomically diverse region.^3^ Approximately 7% of members enroll through Medicaid and 10% through Medicare.

### Selection of Participants

Patients were members of KPSC with at least one ED visit and discharge from January 1, 2009, to December 31, 2010. A patient had to be a member of the health plan at the time of the ED visit; however, no minimum enrollment history was required. Analyses were restricted to adults age ≥ 65 years, as these patients have a greater number of poor outcomes after discharge.^4^,^5^ All patients in the study were discharged from the ED to home or a non-acute care facility such as a nursing home or rehabilitation facility. If patients had multiple ED visits, then only the first visit was included in the analysis.

We exluded patients who left the ED without being seen by a health provider. Patients transferred to observation status from the ED were also excluded, as encounters in this setting could resemble an inpatient admission. Patients receiving hospice care were also excluded, as the goal of this type of care is to provide palliative services rather than prolong life. In addition, patients who were transferred to and from other hospitals were excluded. The small number (<0.1%) of visit records that had potentially erroneous day and time entries resulting in either negative or excessively long ED lengths of stay (LOS) (>48 hours), were also excluded from the analysis.

### Data Sources

The analyses used the Kaiser Permanente Epic-based electronic health record (EHR) (KP HealthConnect) for all variables. The EHR contains records of all member visits to health plan EDs. This system contains past history, mode of arrival, vital signs, staff notes, orders, diagnoses, and test results. Standardized data fields from ED visits provide time-stamps for patient registration, triage, assignment to provider, and disposition order (discharge to home, a care facility, or an inpatient bed). KP HealthConnect was also used to identify the *International Classification of Diseases* (ICD) diagnoses associated with the ED visit.

**Population Health Research Capsule**

What do we already know about this issue?

We know that older white males are at greater risk for poor outcomes after emergency department (ED) discharge, a change in disposition from “admit” to “discharge”, cognitive impairment, systolic blood pressure (SBP) < 120 mmHg, and pulse > 90 beats/min.

What was the research question?

This study developed a risk score for adults experiencing an admission or death/intensive care unit placement within 7 days of ED discharge.

What was the major finding of the study?

Patients at risk for either outcome were: age > 80, body mass index<18.5, SBP < 120 mmHg, pulse >100 bpm, high comorbidities, ED length of stay > 4 hrs, and prior admission.

How does this improve population health?

This information helps ED providers and hospital administrators better manage ED patients.

### Risk Factors

The clinical variables were dichotomized a priori by members of the project team (GZG, MKG, SFG, CAS) based on clinical judgment and prior literature.^6^–^8^ Rather than incorporating all vital signs, two vital signs were chosen for parsimony of the risk score.^9^ For 96% of encounters, patients had at least a single vital sign recorded. For patients with visits with more than one measure for a given vital sign, the vital sign closest to discharge was chosen for the analysis. For extreme values of vital signs that were not compatible with life and most likely a coding error, the vital signs were coded as missing: systolic blood pressure (SBP) <50 or >300, heart rate (HR) < 25 or >225. In addition*,* as the team has shown ED LOS to be a possible risk factor for poor outcomes after discharge, 7 we included ED LOS in the model and defined it as the total time a patient spent in the ED from the time they checked in to ED triage to the time they were discharged from the ED.

### Outcome Measures

The primary outcome was an inpatient admission within seven days of discharge from the ED and the secondary outcome was ICU admission or death over the same time period. The seven-day period was chosen based on frequency results that indicated the highest percentage of admissions occurring within seven days of discharge and also because of its clinical relevance, implications for health policy decisions, and use in previous studies.^6^,^10^,^11^ Information regarding admissions to non-KPSC hospitals was obtained through Kaiser billing data. Deaths were identified using vital statistics data from the California Vital Statistics files linked to Kaiser billing data.

### Analysis

We treated each outcome in the same manner. All patient ED visits were identified over the two years and randomly divided into a derivation sample (75% data) and validation sample (25% data). First, each patient characteristic was assessed for associations with the outcomes in the derivation sample using a Pearson’s chi square test. Then, we included statistically significant variables (p <0.1) in the full logistic regression model for each outcome. To evaluate the accuracy of the final logistic regression models, the study team inspected receiver operating characteristic curves and calculated a C-statistic.^12^

To arrive at the risk score, the study team standardized all coefficient estimates of the model variables by dividing by the smallest variable coefficient. Then, a numeric score (point) was applied to each variable based on the result. All points were rounded to the nearest whole number. Predicted probabilities were calculated for each risk score.^13^ To evaluate the calibration of the scoring system, the study team compared the predicted probability of a given score with the observed probability in both the derivation sample and the validation sample.

In the model for inpatient admission, the initial variables were age, gender, race/ethnicity, smoking, Emergency Severity Index, body mass index (BMI), vital signs of SBP and HR, CCI^14^ (see Appendix for measures), ED LOS, ED visit in week prior, and inpatient admission in week prior the ED visit. Then, the study team omitted the variables not statistically significantly associated with the outcome (p-value >0.1) and arrived at a final model of age, BMI, vital signs of SBP and HR, CCI, ED LOS, and inpatient admission in week prior. For death or ICU admission, the same methodology was used, except that the final model included gender.

## RESULTS

### Characteristics of Study Subjects

As illustrated in the figure, during years 2009–2010, there were 1,552,594 visits among 922,005 patients to KPSC ED facilities. Excluded from the analyses were the following: visits from non-KPSC members; visits with missing gender or birthdate; ages lower than 65 years; patients in hospice care; transfers out of or into the ED; death in the ED; direct admission to an inpatient or observation bed from the ED; and visits other than the first visit. The study cohort contained 104,025 patient visits, of which 4.5% experienced an inpatient admission within seven days of ED discharge and 0.5 % either died or had an ICU admission.

Characteristics of the patients are presented in Table 1 by outcome. The mean age of patients who visited the ED and were discharged was 75.3 years (standard deviation [SD] 7.6), while the mean age for patients with an admission and death/ICU placement respectively was 76.8 years (SD 7.9) and 78.0 years (SD 8.3). The cohort contained slightly more visits by females (57%, n=59,517), as well as White (54%, n=56,052) and Hispanic (22%, n=22,963) patients.

### Main Results

Table 2 presents the risk scores for the two outcomes based on the logistic regression models that were composed. The minimum score a patient could receive was 0 for ages 65–79 years, BMI ≥ 18.5, SBP > 120 mmHg, HR < 100 bpm, no Charlson comorbidities, ED LOS < 5 hours, and no inpatient admission the week prior. The maximum score was 30. In the model predicting death/ICU placement, the minimum score a patient could receive was 0 and the maximum 21. While risk factors were similar for the two outcomes, the scoring was slightly different.

To illustrate the application of the risk score, assume a very thin (with an estimated BMI of 16), 70-year-old male is seen in the ED with a SBP of 110 millimeters mercury (mmHg), HR of 110 beats per minute (bpm), has a history of diabetes and hypertension, stays in the ED for 12 hours, and has not been admitted in the prior seven days. The patient’s risk factors would give him a score of 19 for inpatient admission and 16 for death/ICU placement.

To assess the validity of the risk scores, the predicted as well as the observed probabilities of seven-day admission and seven-day death/ICU placement were assessed (Appendix). Following the numerical predictions are plots and ROC curves for the two models. The C-statistic was 0.68 (for inpatient admission) and 0.76 (for death/ICU placement).

## DISCUSSION

The study identified simple measures that can be used to calculate a risk score for developing a poor outcome after ED discharge. Patients with the greatest likelihood and highest score (score of 40) for developing an inpatient admission within seven days of discharge were age ≥ 80 years old (score of 1), BMI <18.5 (score of 3), SBP ≤120 mm Hg (score of 2), HR ≥ 100 bpm (score of 4), CCI score of 7 or greater (score of 8), ED LOS of 10–24 hours (score of 7), and an inpatient admission in the past seven days (score of 5). Patients at greatest risk for death or an ICU placement (score of 19) were male (score of 1), age ≥ 80 years old (score of 1), BMI <18.5 (score of 3), SBP ≤120 mmHg (Score of 2), HR ≥100 bpm (score of 3), CCI score of 7+ (Score of 5), and ED LOS of 10–24 hours (score of 4).

A low BMI (<18.5) led to greater risk for either outcome. Various studies have found that adults with higher BMIs, either overweight range (BMI ≥ 27.3) or obese (obese ≥ 30), often experience worse outcomes.^15^–^17^ Yet, recent studies suggest that older adults with high BMIs have lower incidences of poor outcomes and that a low BMI could result in worse outcomes.^16^,^18^ The current study results in older adults confirm these findings.

As can be clinically concerning, an SBP below or equal to 120 mmHg and a HR ≥ 100 bpm was associated with a poor outcome after discharge. While these vital signs are markers of hemodynamic instability, they should especially concern an emergency provider.

The study found a high CCI^14^ (>4) to be the greatest predictor (with highest number of points) for both outcomes. Since its publication, the CCI has undergone numerous modifications to conform with recent changes in ICD codes.^19^,^20^ This study indicates that although a specific complaint (i.e., chest pain) requires attention, so too does the past medical history.

While ED LOS, defined as the total time a patient remains in the ED from registration to discharge, can be a marker of ED crowding, it may capture something unrelated to ED crowding about the patient’s complexity and risk for poor outcomes. There have been conflicting results regarding ED LOS and outcomes after discharge, which suggest that this is a complicated measure. A prior study that did not adjust for case-mix severity found a relationship between ED LOS and poor outcomes after discharge.^11^ A study conducted by our project team that did adjust for case mix did not find an association with a poor outcome.^7^ This study found that a prolonged ED LOS past four hours contributes to the risk score (5–9 hours, 2 points) and (10–24 hours, 4 points).

Admission in the past seven days was also found to contribute to developing a poor outcome after discharge (inpatient admission, 5 points; death/ICU placement, 2 points). Older adults have a higher rate of utilization of medical services^21^ and prior studies have attempted to predict hospital utilization following an ED visit.^22^–^25^ Yet there is insufficient evidence to understand whether patients with recent use of hospital services are at greater risk for a poor outcome after ED discharge. This study found that patients with an inpatient hospitalization within the seven days prior to the ED visit have a greater likelihood of a poor outcome after discharge.

Although this study identified patients who sustain poor outcomes after discharge, the study team did not determine whether the outcomes were preventable vs. inevitable. The study team suggests that the risk score components identified compose either an electronic or mental flag for each provider seeing the patient. The provider could then ensure that the patient receives an evaluation prior to discharge or arrange for close follow-up following discharge. In addition, the rate of discharge of patients in this cohort was higher than national averages, which have been found to be in the 40% range.^26^ This could be attributed to the lack of generalizability of the KPSC system as indicated below. It may also affect the rate of admission rates after discharge.

## LIMITATIONS

The study has some limitations. First, as indicated above, the results may not generalize to other settings. KPSC members have access to follow-up care that patients in other settings may lack. KPSC hospitals may also have different disposition courses for patients seen in the ED as a cause of their follow-up options. Second, the admission outcome did not include observation stays. Given the increasing use of observation services, however, future studies should consider incorporating observation stays into admission outcomes. A third limitation inherent to the type of study performed was the lack of available clinical information regarding the chief complaint as well as the extent of management/treatment performed. Also, the reason why patients were admitted or died following discharge from the ED is unknown. Finally, the data used for this analysis are for years 2009–2010; while this is an extended time frame, patients have not changed since then.

## CONCLUSION

This study determined two risk scores for developing a poor outcome following ED discharge in an integrated health system. Patients at greatest risk for either inpatient admission or death/ICU placement within seven days of ED discharge have the following characteristics: age ≥ 80, BMI <18.5, SBP ≤ 120 mmHg, HR ≥100 bpm, high number of comorbidities, ED LOS greater than four hours, and prior inpatient admission in seven days prior to the ED visit.

## Supplementary Information



## Figures and Tables

**Figure:**
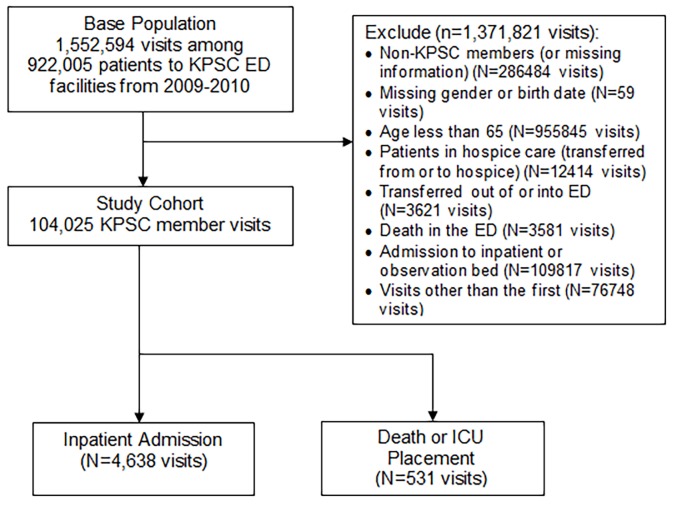
Outline of study cohort.

**Table 1:** Characteristics of the study cohort.

Characteristics	Total N=104,025	Patients w/admission N=4,638 (%)	P value*	Patients w/death or ICU placement N=531 (%)	P value*
Age (mean, SD)	75.3 (7.6)	76.8 (7.9)	< 0.0001	78.0 (8.3)	< 0.0001
Age			< 0.0001		< 0.0001
65–79	73845	2975 (4.0%)		312 (0.4%)	
80+	30180	1663 (5.5%)		219 (0.7%)	
Gender			0.0008		< 0.0001
Male	44508	2095 (4.7%)		275 (0.6%)	
Female	59517	2543 (4.3%)		256 (0.4%)	
Race			< 0.0001		< 0.0001
White	56052	2791 (5.0%)		305 (0.5%)	
Black	14349	585 (4.1%)		73 (0.5%)	
Hispanic	22963	920 (4.0%)		101 (0.4%)	
Asian	8354	319 (3.8%)		47 (0.6%)	
Other	2307	23 (1.0%)		5 (0.2%)	
BMI			< 0.0001		< 0.0001
< 18.5	2077	163 (7.8%)		36 (1.7%)	
18.5+	101948	4475 (4.4%)		495 (0.5%)	
Charlson index			< 0.0001		< 0.0001
0	20335	449 (2.2%)		32 (0.2%)	
1	18176	515 (2.8%)		39 (0.2%)	
2	14901	554 (3.7%)		64 (0.4%)	
3	13369	590 (4.4%)		66 (0.5%)	
4	10276	533 (5.2%)		61 (0.6%)	
5	7621	448 (5.9%)		44 (0.6%)	
6	6441	441 (6.8%)		62 (1.0%)	
7+	12906	1108 (8.6%)		163 (1.3%)	
Vital signs					
SBP			< 0.0001		< 0.0001
≤ 120	19726	1224 (6.2%)		185 (0.9%)	
> 120	84299	3414 (4.0%)		346 (0.4%)	
HR			< 0.0001		< 0.0001
< 100	99760	4294 (4.3%)		458 (0.5%)	
≥ 100	4265	344 (8.1%)		73 (1.7%)	
Length of stay			< 0.0001		< 0.0001
0–4 hrs	78774	2734 (3.5%)		301 (0.4%)	
5–9 hrs	22967	1671 (7.3%)		193 (0.8%)	
10–24 hrs	2284	233 (10.2%)		37 (167%)	
Admission**			< 0.0001		< 0.0001
Y	2734	289 (10.6%)		44 (1.6%)	
N	101291	4349 (4.3%)		487 (0.5%)	

* P-value is generated using chi square analysis.

** Inpatient admission in past seven days.

*ICU*, intensive care unit; *SD*, standard deviation; *BMI*, body mass index; *SBP*, systolic blood pressure; *HR*, heart rate.

**Table 2:** Risk scores.

Score for inpatient admission		Score for death/ICU placement	
	
Risk factor	Score	Risk factor	Score
		Gender (Male)	1
Age 80+	1	Age 80+	1
BMI < 18.5	3	BMI < 18.5	3
SBP ≤ 120	2	SBP ≤ 120	2
Pulse ≥ 100	4	Pulse ≥ 100	3
Charlson score		Charlson score	
1	1	1	1
2	3	2	3
3	4	3	3
4	5	4	3
5	6	5	3
6	7	6	4
≥7	8	7+	5
Length of stay		Length of stay	
5–9 hrs	4	5–9 hrs	2
10–24 hrs	7	10–24 hrs	4
Inpatient 7 (Yes)	5	Inpatient 7 (Yes)	2

*ICU*, intensive care unit; *BMI*, body mass index; *SBP*, systolic blood pressure.

This table presents the risk scores for the two outcomes. For inpatient admission, the minimum score a patient could receive was 0 and the maximum 30. For the death/ICU placement, the minimum score was 0 and maximum score was 21.
