# Supplementary material for: A Risk Score to Predict Short-term Outcomes Following Emergency Department Discharge
Source: West J Emerg Med. 2018 Aug 13;19(5):842–8. doi: 10.5811/westjem.2018.7.37945 (PMC6123082; doi:10.5811/westjem.2018.7.37945)
Supplement: Supplementary file 1 [file wjem-19-842-s001.docx]

**Supplementary Section**

**A: Predicted vs. Observed Risk**

1. For inpatient admission within 7 days of discharge

|  |  | Derivation Sample | | Validation Sample | |
| --- | --- | --- | --- | --- | --- |
| Risk Score | Predicted Risk | Sample Size (N) | Observed Risk | Sample Size (N) | Observed Risk |
| 0 | 0.0165 | 8111 | 0.0123 | 2798 | 0.0114 |
| 1 | 0.0191 | 8124 | 0.0171 | 2690 | 0.0230 |
| 2 | 0.0223 | 3141 | 0.0232 | 1117 | 0.0179 |
| 3 | 0.0259 | 6549 | 0.0221 | 2073 | 0.0188 |
| 4 | 0.0301 | 8257 | 0.0309 | 2738 | 0.0278 |
| 5 | 0.0350 | 8028 | 0.0349 | 2646 | 0.0363 |
| 6 | 0.0407 | 5711 | 0.0473 | 1919 | 0.0396 |
| 7 | 0.0473 | 5911 | 0.0502 | 1946 | 0.0540 |
| 8 | 0.0550 | 6806 | 0.0619 | 2269 | 0.0520 |
| 9 | 0.0640 | 5074 | 0.0609 | 1674 | 0.0687 |
| 10 | 0.0744 | 3235 | 0.0791 | 1072 | 0.0840 |
| 11 | 0.0865 | 2439 | 0.0791 | 800 | 0.0863 |
| 12 | 0.1006 | 2420 | 0.093 | 845 | 0.0923 |
| 13 | 0.1170 | 1686 | 0.0991 | 551 | 0.1053 |
| 14 | 0.1361 | 930 | 0.1183 | 333 | 0.1231 |
| 15 | 0.1583 | 658 | 0.1444 | 228 | 0.1096 |
| 16 | 0.1840 | 377 | 0.1406 | 114 | 0.1491 |
| 17 | 0.2140 | 231 | 0.1385 | 71 | 0.2113 |
| 18 | 0.2489 | 160 | 0.1313 | 58 | 0.1207 |
| 19 | 0.2894 | 80 | 0.2375 | 26 | 0.1923 |
| 20+ | 0.2868 | 92 | 0.2826 | 37 | 0.2162 |

1. For death or an ICU placement within 7 days of discharge

|  |  | Derivation Sample | | Validation Sample | |
| --- | --- | --- | --- | --- | --- |
| Risk Score | Predicted Risk | Sample Size | Observed Risk | Sample Size | Observed Risk |
| 0 | 0.0008 | 4797 | 0.0004 | 1659 | 0.0006 |
| 1 | 0.0011 | 8328 | 0.0008 | 2763 | 0.0004 |
| 2 | 0.0015 | 6195 | 0.0010 | 2107 | 0.0028 |
| 3 | 0.0022 | 11544 | 0.0024 | 3812 | 0.0018 |
| 4 | 0.0031 | 13696 | 0.0031 | 4487 | 0.0031 |
| 5 | 0.0044 | 10144 | 0.0042 | 3325 | 0.0063 |
| 6 | 0.0063 | 9440 | 0.0067 | 3245 | 0.0080 |
| 7 | 0.0090 | 5904 | 0.0080 | 1889 | 0.0090 |
| 8 | 0.0128 | 3788 | 0.0111 | 1310 | 0.0115 |
| 9 | 0.0182 | 2115 | 0.0189 | 721 | 0.0166 |
| 10 | 0.0260 | 1038 | 0.0318 | 340 | 0.0147 |
| 11 | 0.0370 | 633 | 0.0284 | 209 | 0.0239 |
| 12 | 0.0528 | 222 | 0.0450 | 70 | 0.0143 |
| 13 | 0.0752 | 108 | 0.0370 | 41 | 0.0732 |
| 14 | 0.1071 | 43 | 0.0930 | 17 | 0.1176 |
| 15+ | 0.1685 | 25 | 0.1600 | 10 | 0.2000 |

B: Pictorial Representation of predicted vs. observed probability

1. For outcome of inpatient admission


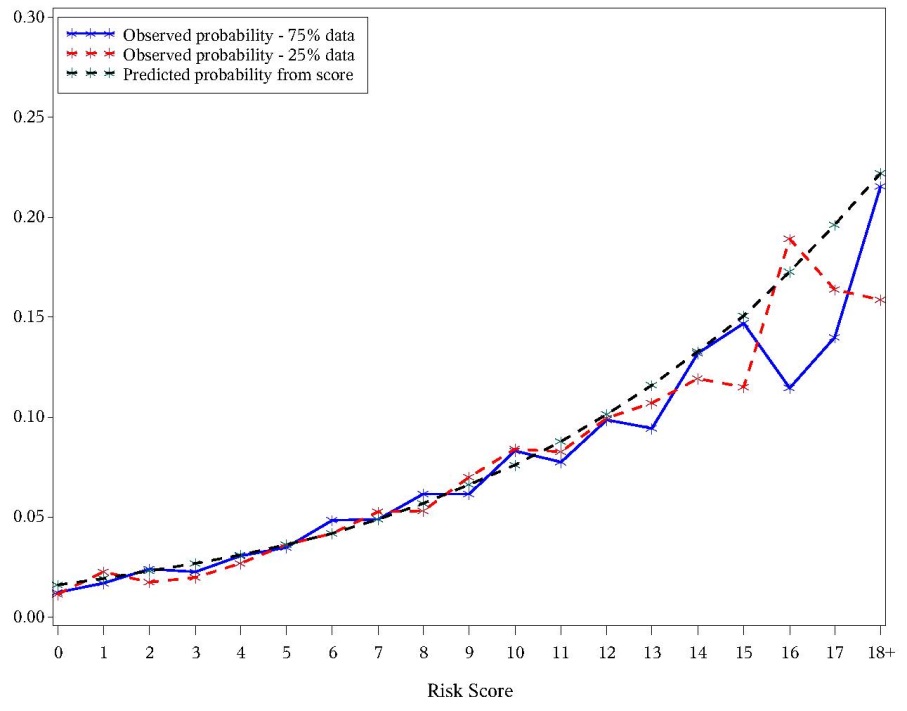


1. For outcome of death or an ICU placement


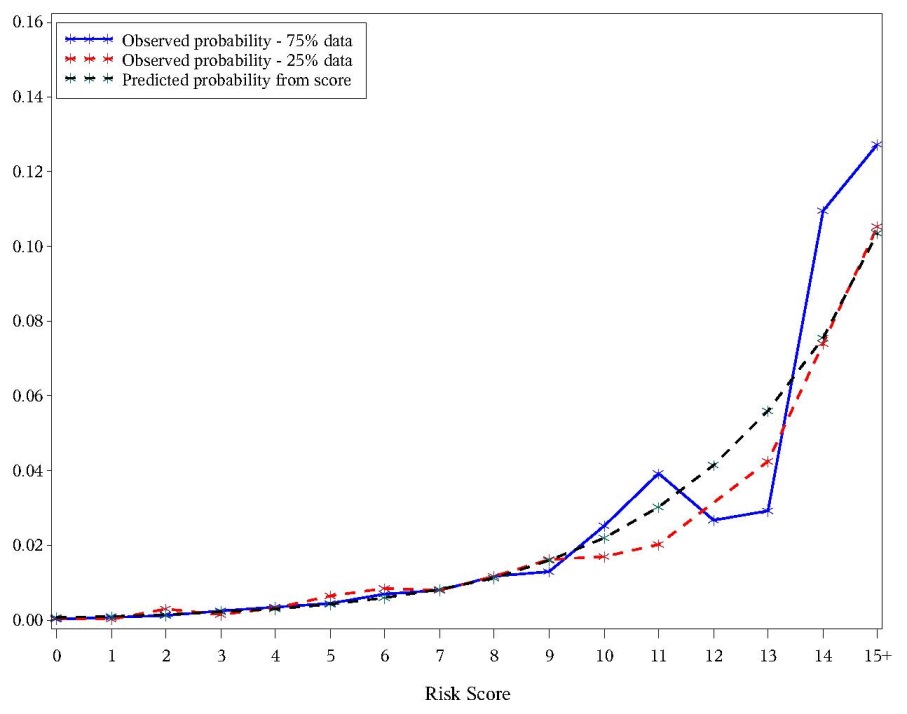


**C: ROC Curves**

1. ROC Curve for the model predicting inpatient admission


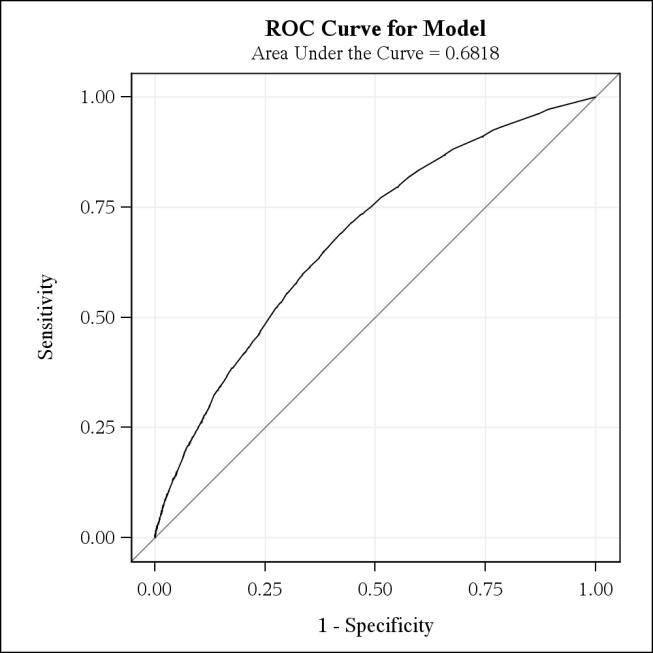


1. ROC Curve for the model predicting death or ICU placement


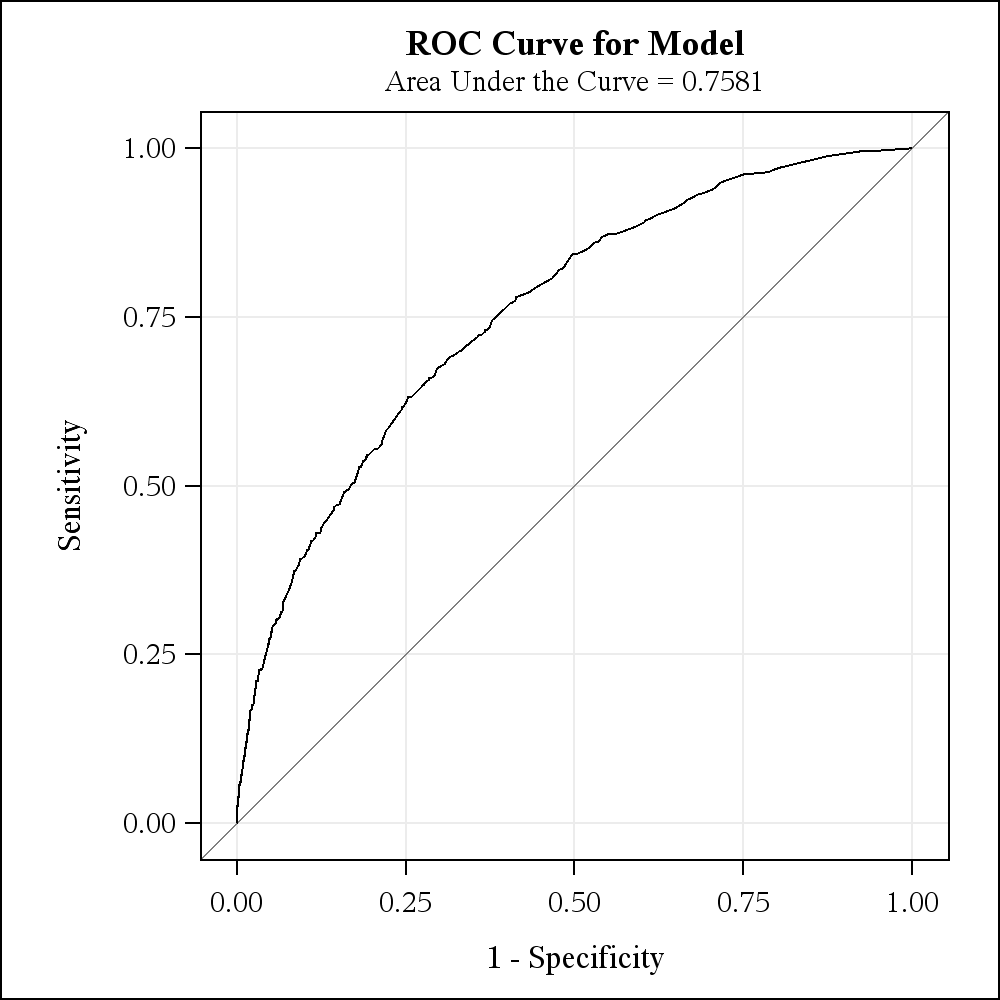


**D: Charlson Comorbidity Index Measures**

| Myocardial infarction |
| --- |
| Congestive heart failure |
| Peripheral vascular  disease |
| Cerebrovascular disease |
| Dementia |
| Chronic pulmonary  disease |
| Rheumatic disease |
| Peptic ulcer disease |
| Mild liver disease |
| Diabetes without chronic  complication |
| Diabetes with chronic  complication |
| Hemiplegia or paraplegia |
| Renal disease |
| Any malignancy,  including lymphoma  and leukemia, except  malignant neoplasm of skin |
| Moderate or severe liver  disease |
| Metastatic solid tumor |
| AIDS/HIV |
